# Exploration of the Relationship Among Key Risk Factors of Acute Kidney Injury for Elderly Patients Considering Covid-19

**DOI:** 10.3389/fmed.2021.639250

**Published:** 2021-07-22

**Authors:** Yen-Ching Chuang, Tao-Hsin Tung, Jau-Yuan Chen, Ching-Wen Chien, Kao-Yi Shen

**Affiliations:** ^1^Institute of Public Health & Emergency Management, Taizhou University, Taizhou, China; ^2^Taizhou Hospital of Zhejiang Province Affiliated to Wenzhou Medical University, Taizhou, China; ^3^Department of Family Medicine, Linkou Chang-Gung Memorial Hospital and Chang-Gung University, Taoyuan, Taiwan; ^4^Institute for Hospital Management, Tsing Hua University, Shenzhen Campus, Shenzhen, China; ^5^Department of Banking and Finance, Chinese Culture University, Taipei, Taiwan

**Keywords:** elderly, frailty, acute kidney injury (AKI), risk assessment framework, influential network-relation structure, coronavirus disease 2019 (Covid-19), decision-making trial and evaluation laboratory (DEMATEL), multiple criteria decision-making (MCDM)

## Abstract

**Background:** Previous systematic reviews and meta-analyses supported the relationship between frailty and risk of acute kidney injury (AKI) in elderly patients. However, few studies evaluated proactive management to wear down AKI risk in such frail populations.

**Purpose:** To understand how AKI risk factors might influence each other and to identify the source factors for clinical decision aids.

**Methods:** This study uses the decision-making trial and evaluation laboratory (DEMATEL) method to establish influential network-relationship diagrams (INRDs) to form the AKI risk assessment model for the elderly.

**Results:** Based on the DEMATEL approach, the results of INRD identified the six key risk factors: comorbidity, malignancy, diabetes, creatinine, estimated glomerular filtration rate, and nutritional assessment. (The statistical significance confidence is 98.423%, which is higher than 95%; the gap error is 1.577%, which is lower than 5%). After considering COVID-19 as an additional risk factor in comorbidity, the INRD revealed a similar influential relationship among the essential aspects.

**Conclusion:** While evaluating the geriatric population, physicians need to pay attention to patients' comorbidities and nutritional assessment; also, they should note patients' creatinine values and glomerular filtration rate. Physicians could establish a preliminary observation index and then design a series of preventive guidelines to reduce the incidence of AKI risk for the elderly.

## Introduction

Acute kidney injury (AKI) is a medical complication with a high risk of morbidity and mortality (1, 2), especially for elderly patients. Since older age has been regarded as an AKI risk factor (3) and most developed countries have increased aging populations (4), there has been a surging interest in the relationship between aging and AKI. Compared to younger patients, elderly AKI patients are prone to worse kidney recovery and higher mortality (5, 6). Therefore, some studies have explored the predictive factors of AKI for elderly patients, in an attempt to diagnose or mitigate AKI risk during the early stage (3, 7–11).

The recent global outbreak of coronavirus disease 2019 (COVID-19) has imposed additional threats to the elderly, who are more vulnerable to this pandemic (12, 13). Although, most patients with Covid-19 have mild symptoms, elderly patients are more likely to develop severe symptoms, such as acute respiratory distress and multiple organ failure, or even death. Kidney-related symptoms are frequent, and this circumstance has increased AKI risk for elderly. According to Ronco et al. (14), there are no specific treatment options for AKI secondary to COVID-19 at this moment. How to prevent and manage AKI risk for elderly patients mainly depends on clinical experience. Therefore, the present study also attempts to investigate the influence of COVID-19 on AKI risk for elderly patients.

Though, various factors may cause AKI, frailty, a physiological decline syndrome associated with aging, is a particular issue in geriatric populations, Frailty increases the health risk of elderly patients (15–18) and has emerged as a predictive factor of adverse outcomes for elderly patients (19). A previous study adopted frailty as a predictor of AKI in hospitalized elderly patients (1); its results indicated that, during hospitalization, frailty might predict the elderly patients' development and adverse outcome of AKI. Another prospective cohort study revealed similar findings; it showed that the association between “severe frailty” and AKI is significantly higher for elderly patients (20). Recently, a meta-analysis study confirmed the association between frailty and AKI in elderly patients (21). While most evidence-based studies seem to support the association between frailty and AKI for the elderly, the relationship among crucial AKI risk factors is still unclear.

Thus, this study constructed two influential network-relationship diagrams (INRDs) to explore the relationship among AKI risk factors, with or without COVID-19, to fill the gap. The included factors (attributes) were based on previous studies (2, 18) and the opinions from a small group of nephrologists in Taiwan. The two INRDs reveal the influential relationship among AKI risk factors, analyzed using decision-making trial and evaluation laboratory (DEMATEL) technique. The findings contribute to the understanding of how AKI risk factors might influence each other and help to identify the source factors for clinical decision aids. The main findings of this study are as follows:

1. This study summarized the crucial factors that might lead to AKI for older patients based on previous research and the opinions from 10 experienced nephrologists;
2. The influential relationship among the critical aspects and the associated attributes (factors) are clarified and illustrated in **Figure 2** (both *Comorbidity* and *Laboratory values* would influence *Comprehensive geriatric assessment*);
3. The influence of COVID-19 on AKI for elderly patients was analyzed by the proposed DEMATEL technique (**Figure 3**), which is similar to the one without COVID-19;
4. All the included Comorbidity diseases (e.g., hypertension) would influence the consequence brought by COVID-19 to elderly patients.

The remainder of this study is structured as follows: Materials and Methods introduces the risk assessment framework regarding AKI for elderly patients, and describes the history and calculation procedures of the DEMATEL technique. Research design illustrates the study design of this research. In Results, we demonstrate how to apply the DEMATEL technique and obtain the two INRDs. Discussion discusses the findings and concludes this study with future research directions.

## Materials and Methods

### The AKI Risk Assessment Framework for Elderly Patients

In this study, the standard for elderly patients is over 65 years old. The AKI assessment framework is based on meta-analysis research (21) and its associated studies (1, 2, 9, 20, 22–28) to form a pool of risk factors, including the criteria associated with geriatric assessment. In this context, we followed the definition of frailty from the meta-analysis article (21). This meta-analysis identified 1,096 articles after removing duplicates. Eventually, four publications reporting four cohort studies with 1,052 study subjects met the inclusion criteria. The selected studies were published between 2016 and 2018. All four cohort studies were population-based cohort studies from the UK, the USA, and South Korea. Furthermore, all selected studies were considered with high quality and low-to-moderate risk using ROBINS-I (21).

In addition, the estimated glomerular filtration rate (eGFR) is based on the CKD epidemiology collaboration formula (29). However, the creatinine (*C*_21_) and the estimated glomerular filtration rate (*C*_22_) are somewhat similar. From a clinical point of view, this model may reveal some practical significance of the independent effects of creatinine and estimated glomerular filtration rate (*C*_22_). Therefore, creatinine (*C*_21_) is retained in this study under this consideration. Next, after removing the demographic variables, we had several rounds of discussions with the doctors to identify 13 AKI risk factors, summarized in Table 1.

**Table 1:** The AKI risk assessment framework.

**Aspects**	**Attributes**	**References**
Comorbidity (*C*_1_)	Diabetes (*C*_11_)	(1, 2, 20, 28)
	Hypertension (*C*_12_)	(1, 2, 20)
	Depression (*C*_13_)	(9, 25)
	Malignancy (*C*_14_)	(1, 2)
Laboratory values (*C*_2_)	Creatinine (*C*_21_)	(1)
	Estimated glomerular filtration rate (*C*_22_)	(1)
	Hemoglobin (*C*_23_)	(1)
	Albumin (*C*_24_)	(1)
	Na (*C*_25_)	(1)
Comprehensive geriatric assessment (*C*_3_)	Activities of daily living (*C*_31_)	(1)
	Mid-arm circumference (*C*_32_)	(1)
	Frailty (*C*_33_)	(1, 20, 22, 23)
	Nutritional assessment (*C*_34_)	(1)

### The DEMATEL Method

The DEMATEL method was developed by Battelle Memorial Institute in 1972 for solving interdependent structure problems in the real world (30). The method was built on the foundation of graph theory, enabling analysis and solving problems with a visualization method (31). Hence, this structural modeling method can help decision-makers better understand the interdependent relationship among elements, and find various cause-effect ways to solve complicated system problems. For these reasons, the improvement strategy with cause-effect relationship can let the decision-makers know that it has a set of systemic perspective policies, meaning they do not have to pursue a piecemeal method (31). Therefore, the DEMATEL method has been one of the most popular structural modeling methods and has been successfully applied in various fields, such as investment projects (32), digital platforms (33), green roofs (34), cloud services (35), green suppliers (36), green building (37), University teaching (31), and sustainable education environments (38). The calculation steps and description are as follows (36, 39):

Step 1: Building an initial influence relation matrix.

An evaluation system with *n* indicators/attributes is confirmed. Each nephrologist/expert fills in the degree of interdependent relation between attributes, through the five-point Liker scale [no influence (0) to very high influence (4)]. Each expert draws a matrix ***A*** = [_*a*_*io*_]*n×n*_ of direct influence relation based on his/her clinical experience. Finally, these direct influence relation matrixes can be integrated into a matrix, namely the initial influence relation matrix, as shown in Equation (1).

(1) $$\begin{array}{l} S={\left[s_{io}\right]}_{n\times n}={\left[\left(\sum_{\varphi =1}^{k}a_{io}^{\varphi }\right)/k\right]}_{n\times n} \end{array}$$

where ***S*** is the initial influence relation matrix in which all principal diagonal elements are equal to zero.

Step 2: Obtaining an normalized influence relation matrix ***D***.

The significance of this step is that the next step can be based on the Markov chain process to obtain the degree of multiple influence relations (38, 39). The normalized influence relation matrix ***D*** can be derived from the initial influence relation matrix ***S*** through Equations (2) and (3).

(2) $$\begin{array}{lll} D & = & \frac{S}{\Theta } \end{array}$$

(3) $$\begin{array}{lll} \Theta & = & \underset{i,o}{\max}\left\{\max_{i}\sum_{o=1}^{n}s_{io},\max_{o}\sum_{i=1}^{n}s_{io}\right\},i,o\in \left\{1,2,\ldots ,n\right\} \end{array}$$

where the maximum sum of each row or column is one in the matrix ***D***.

Step 3: Producing a total influence relation matrix ***T***.

The normalized influence relation matrix ***D*** calculates the degree of total influence relationship between attributes through Equation (4), and finally obtains the total influence relationship matrix ***T***.

(4) $$\begin{array}{l} T\;\text{=}\;D\text{+}D^{2}\text{+}\ldots \text{+}D^{h}=D{\left(I-D\right)}^{-1},\text{when}\underset{h\to \infty }{\lim}D^{h}={\left[0\right]}_{n\times n} \end{array}$$

where the matrix ***I*** is the identify matrix.

Step 4: Drawing an influential network-relation diagram (INRD).

The total influence relationship matrix ***T*** uses Equations (5) and (6) to obtain *p*_*i*_ and *y*_*i*_, respectively. The former (*p*_*i*_) represents the total influence degree of attribute *i* on other attributes; the latter (*y*_*i*_) represents the total influence degree of other attributes on attribute *i*. These two variables are also called given (*p*_*i*_) and received (*y*_*i*_), respectively.

(5) $$\begin{array}{l} p_{i}={\left(p_{i}\right)}_{n\times 1}=\left(p_{1},...,p_{i},...,p_{n}\right)={\left[\sum_{o=1}^{n}t_{io}\right]}_{n\times 1} \end{array}$$

(6) $$\begin{array}{l} y_{i}={\left(y_{i}\right)}_{n\times 1}={{\left(y_{o}\right)}^{\prime }}_{1\times n}={\left(y_{1},...,y_{o},...,y_{n}\right)}^{\Gamma }={\left[\sum_{i=1}^{n}t_{io}\right]}_{1\times n}^{\Gamma } \end{array}$$

where the symbol Γ denotes the transpose action.

The variables of given (*p*_*i*_) and received (*y*_*i*_) can be produced into the other two variables regarding the “prominence (*p*_*i*_ + *y*_*i*_)” and the “relation (*p*_*i*_ − *y*_*i*_).” The “prominence (*p*_*i*_ + *y*_*i*_)” denotes the central role of attribute *i* in the evaluation system. The “relation (*p*_*i*_ − *y*_*i*_)” denotes the main influence nature of attribute *i* in the evaluation system. If “relation (*p*_*i*_ − *y*_*i*_)” is positive, attribute *i* belongs to the cause group in the evaluation system (i.e., the influence of attribute *i* mainly affects other attributes). On the contrary, if “relation (*p*_*i*_ − *y*_*i*_)” is negative, attribute *i* belongs to the cause group in the evaluation system (i.e., the influence of attribute *i* mainly affected by other attributes).

Finally, “prominence (*p*_*i*_ + *y*_*i*_)” and the “relation (*p*_*i*_ − *y*_*i*_)” are used as the x-axis and y-axis of the influential network relationship diagram (INRD), respectively, and the influence relationship between attributes in the entire evaluation system can be visualized. According to the results of INRD, nephrologists/decision-makers can understand the mutual influence between attributes, and further analyze the key factors derived from all attributes based on a systematic perspective.

## Research Design

**Figure 1:**
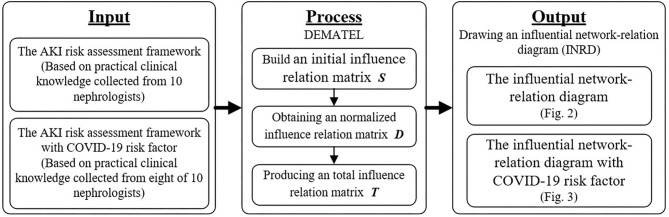
The research flow chart.

The DEMATEL method can estimate the influence-relation between attributes systematically and can help decision-makers identify the most critical attributes from limited attributes. We also took a two-step approach to derive the AKI risk factors for this study. First, the AKI assessment framework is based on the meta-analysis research (21) and its associated studies (1, 2, 9, 20, 22–28). Then, the DEMATEL method is used to construct the interdependence among risk factors. Also, to explore the plausible influence of COVID-19 for elderly patients, we added COVID-19 as an additional risk factor to conduct another DEMATEL analysis. The process of design and analysis of this study is shown in Figure 1.

### Data Collection

The survey data is based on practical clinical knowledge collected from 10 nephrologists who understand the research topic in the elderly population (the statistical significance confidence is 98.423%, which is always higher than 95%; the gap error is 1.577%, which is lower than 5%). The average experience of experts is between 10 and 15 years. The opinions of all experts on the relationship between attributes are collected through questionnaire surveys and personal interviews. An expert survey was conducted in April 2020, and each expert took an average of 40 to 50 min to complete them. The initial influence-relation matrix *S* is shown in Table 2.

**Table 2:** The initial influence-relationship matrix.

	***C*_**11**_**	***C*_**12**_**	***C*_**13**_**	***C*_**14**_**	***C*_**21**_**	***C*_**22**_**	***C*_**23**_**	***C*_**24**_**	***C*_**25**_**	***C*_**31**_**	***C*_**32**_**	***C*_**33**_**	***C*_**34**_**
*C*_11_	–	3.500	2.700	2.500	3.400	3.400	2.300	2.300	2.100	2.800	2.500	2.600	2.300
*C*_12_	2.300	–	1.300	1.600	3.500	3.500	1.600	2.200	2.300	2.100	1.900	2.000	1.900
*C*_13_	1.900	1.700	–	1.700	1.500	1.500	1.200	1.800	1.100	3.600	2.700	3.200	3.000
*C*_14_	2.000	1.900	3.600	–	2.700	2.600	3.500	3.200	2.800	3.700	3.300	3.800	3.800
*C*_21_	1.100	2.000	1.000	1.000	–	3.900	2.900	2.600	3.300	3.300	3.000	3.400	3.300
*C*_22_	1.200	2.200	1.600	2.300	4.000	–	2.900	3.100	3.300	3.500	3.000	3.400	3.300
*C*_23_	0.900	1.300	1.300	1.100	1.800	1.800	–	2.600	1.000	3.000	2.600	3.000	2.500
*C*_24_	1.000	1.000	1.000	1.000	1.400	1.400	1.300	–	1.800	3.000	3.300	3.000	3.600
*C*_25_	0.900	2.500	0.800	1.000	2.800	2.800	1.000	1.200	–	2.000	1.100	2.000	2.100
*C*_31_	3.300	3.300	3.700	2.900	1.800	1.800	2.000	2.000	1.900	–	3.400	3.600	3.600
*C*_32_	1.700	1.700	1.800	1.800	2.100	2.100	2.000	2.000	1.700	2.300	–	3.500	3.500
*C*_33_	2.100	2.000	3.600	2.400	2.100	2.100	2.000	2.200	1.800	3.700	3.500	–	3.700
*C*_34_	3.100	3.000	2.700	2.800	2.600	2.600	2.700	2.900	2.800	3.800	3.900	3.700	–

*The significant confidence equation is $\frac{1}{n\left(n-1\right)}\overset{\varphi }{\underset{i=1}{\sum }}\overset{\varphi }{\underset{o=1}{\sum }}\frac{\left|a_{io}^{\varphi }-a_{io}^{\varphi -1}\right|}{a_{io}^{\varphi }}\times 100\%=1.\text{577}\%<5\%$, i.e., significant confidence is 98.423%, where φ = 10 denotes the number of influential strength matrixes and $a_{io}^{\varphi }$ is the average influence of indicator/attribute i on o; and n denotes the number of indicators, where n = 13*.

## Results

The initial influence relationship matrix ***S*** (Table 2) applies Equations (2–4) to calculate the degree of influence relationship among attributes. Then, we can obtain a matrix, namely the total influence-relationship matrix ***T***, shown in Table 3. The total influence relationship matrix ***T*** (Table 3) can be transformed to get *p*_*i*_ and *y*_*i*_ of each aspect and attribute, referring to Equations (5) and (6), and leads to the “Prominence (*p*_*i*_ + *y*_*i*_)” and “Relation (*p*_*i*_ − *y*_*i*_)” values, respectively.

**Table 3:** The total influence-relationship matrix.

	***C*_**11**_**	***C*_**12**_**	***C*_**13**_**	***C*_**14**_**	***C*_**21**_**	***C*_**22**_**	***C*_**23**_**	***C*_**24**_**	***C*_**25**_**	***C*_**31**_**	***C*_**32**_**	***C*_**33**_**	***C*_**34**_**
*C*_11_	0.194	0.315	0.290	0.261	0.338	0.337	0.279	0.301	0.280	0.384	0.360	0.383	0.373
*C*_12_	0.216	0.187	0.215	0.203	0.296	0.295	0.223	0.255	0.245	0.310	0.292	0.312	0.306
*C*_13_	0.206	0.226	0.182	0.204	0.238	0.237	0.207	0.238	0.207	0.339	0.306	0.334	0.326
*C*_14_	0.270	0.304	0.341	0.223	0.348	0.344	0.334	0.351	0.321	0.445	0.417	0.452	0.448
*C*_21_	0.213	0.267	0.237	0.215	0.239	0.333	0.281	0.294	0.296	0.378	0.356	0.385	0.380
*C*_22_	0.232	0.291	0.271	0.262	0.358	0.259	0.301	0.327	0.315	0.410	0.382	0.414	0.408
*C*_23_	0.166	0.198	0.197	0.173	0.226	0.225	0.159	0.240	0.188	0.302	0.282	0.306	0.292
*C*_24_	0.170	0.193	0.191	0.172	0.218	0.217	0.194	0.175	0.209	0.302	0.299	0.307	0.318
*C*_25_	0.150	0.211	0.165	0.155	0.236	0.235	0.170	0.189	0.148	0.253	0.221	0.256	0.256
*C*_31_	0.285	0.318	0.325	0.279	0.306	0.305	0.278	0.300	0.279	0.324	0.391	0.417	0.413
*C*_32_	0.206	0.233	0.233	0.212	0.262	0.260	0.234	0.252	0.230	0.319	0.248	0.351	0.348
*C*_33_	0.245	0.273	0.309	0.255	0.297	0.295	0.265	0.291	0.263	0.396	0.376	0.311	0.397
*C*_34_	0.295	0.330	0.318	0.292	0.346	0.345	0.313	0.342	0.320	0.443	0.427	0.446	0.352

Table 4 shows four indicators, *p*_*i*_, *y*_*i*_, *p*_*i*_ + *y*_*i*_, and *p*_*i*_ − *y*_*i*_, to depict the influential relationship among the AKI risk assessment model's risk factors. The two layers of this model are the aspect's and the attribute's levels. At the aspect's level, the “comorbidity (*C*_1_)” belongs to the cause group and the “laboratory values (*C*_2_)” and “comprehensive geriatric assessment (*C*_3_)” to the effect group. In Table 4, two patterns emerge: (1) the “comorbidity (*C*_1_)” aspect would influence both the “laboratory values (*C*_2_)” and “comprehensive geriatric assessment (*C*_3_)” aspects; (2) the “laboratory values (*C*_2_)” of an elder patient might improve or deteriorate his/her “comprehensive geriatric assessment (*C*_3_)” state.

**Table 4:** The influential indicators regarding aspects and attributes.

**Aspects/attributes**	**Given (*p*_*i*_)**	**Received (*y*_*i*_)**	**Prominence (*p*_*i*_ + *y*_*i*_)**	**Relation (*p*_*i*_ − *y*_*i*_)**	**Group**
Comorbidity (*C*_1_)	0.885	0.722	1.607	0.163	Cause
Diabetes (*C*_11_)	4.093	2.847	6.939	1.246	Cause
Hypertension (*C*_12_)	3.355	3.346	6.702	0.009	Cause
Depression (*C*_13_)	3.250	3.273	6.523	−0.024	Effect
Malignancy (*C*_14_)	4.599	2.905	7.505	1.694	Cause
Laboratory values (*C*_2_)	0.773	0.814	1.587	−0.041	Effect
Creatinine (*C*_21_)	3.872	3.707	7.579	0.165	Cause
Estimated glomerular filtration rate (*C*_22_)	4.230	3.686	7.916	0.544	Cause
Hemoglobin (*C*_23_)	2.956	3.239	6.195	−0.283	Effect
Albumin (*C*_24_)	2.964	3.555	6.518	−0.591	Effect
Na (*C*_25_)	2.644	3.302	5.947	−0.658	Effect
Comprehensive geriatric assessment (*C*_3_)	0.937	1.059	1.996	−0.122	Effect
Activities of daily living (*C*_31_)	4.219	4.605	8.824	−0.386	Effect
Mid-arm circumference (*C*_32_)	3.386	4.357	7.743	−0.971	Effect
Frailty (*C*_33_)	3.974	4.673	8.647	−0.699	Effect
Nutritional assessment (*C*_34_)	4.570	4.616	9.185	−0.046	Effect

*In the value of group, the “cause” represents the aspect/attribute that primarily affects other aspects/attributes. Otherwise, the “effect” represents the aspect/attribute that primarily affected from other aspects/attributes*.

**Figure 2:**
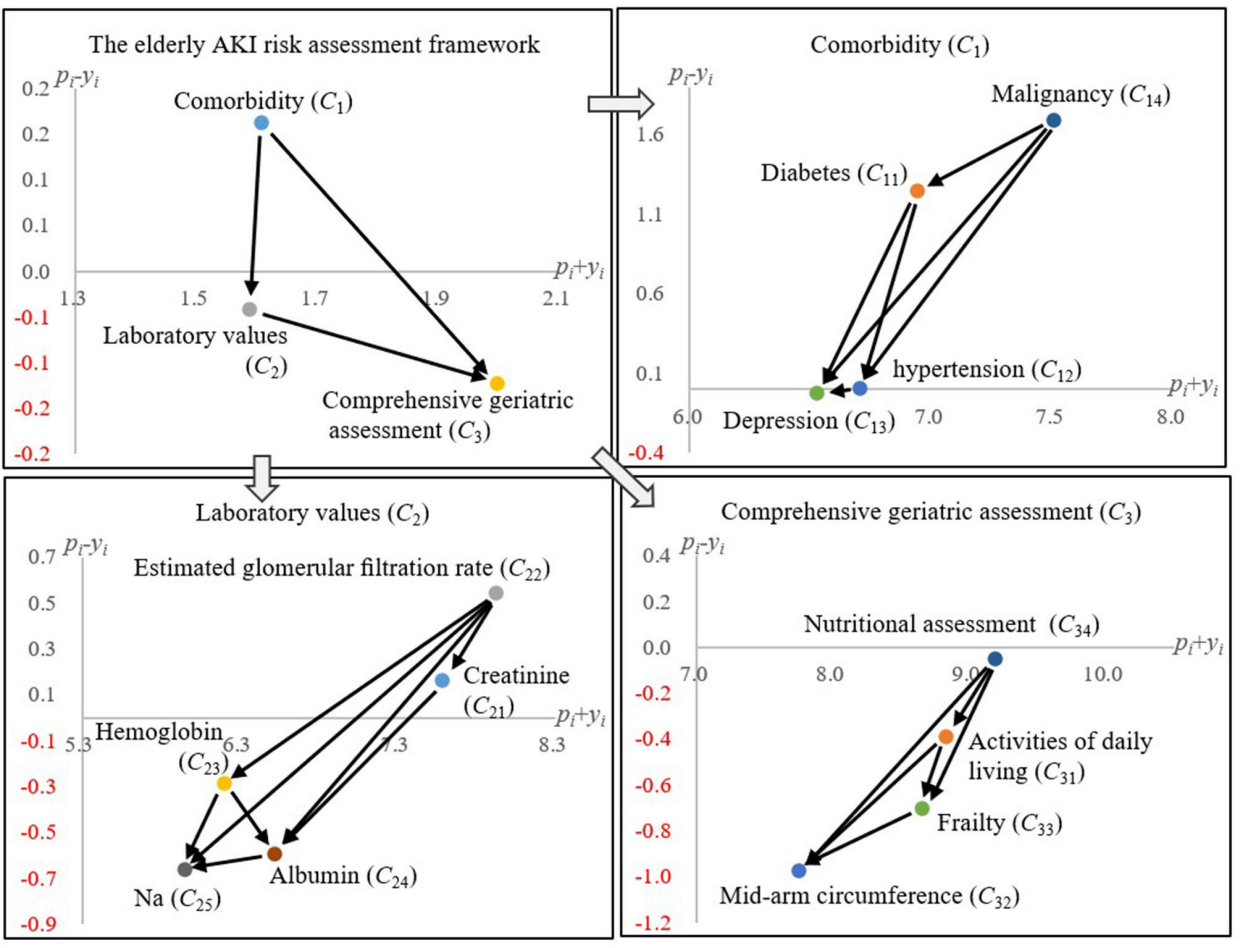
The influential network-relation diagram.

To delve into the attribute level of each aspect, Figure 2 discloses the relationship among the risk factors within an aspect. For instance, in the “comorbidity (*C*_1_)” aspect, it has shown the directional influences among elderly patients' AKI risk factors. Usually, before AKI occurs, elderly patients are hospitalized for other diseases, such as malignancy. From previous research, it is known that the incidence of comorbidities increases significantly with age (40). Furthermore, “malignancy (*C*_14_)” and “diabetes (*C*_11_)” are the source factors that might lead to chronic diseases (i.e., “hypertension (*C*_12_)” and “depression (*C*_13_)”) and increase the AKI risk level.

For the “laboratory values (*C*_2_)” aspect, the “creatinine (*C*_21_)” and “estimated glomerular filtration rate (*C*_22_)” are routine clinical care inspections used to determine the status of AKI (22, 41). Those two attributes are critical AKI risk factors that might influence the others (i.e., *C*_23_, *C*_24_, and *C*_25_) in this aspect. Last, in the “comprehensive geriatric assessment (*C*_3_)” aspect, “nutritional assessment (*C*_34_)” is identified as the crucial source risk factor. The elderly patients with AKI risk would be influenced by daily living activities, inferior cognitive function, and nutritional status (1). In other words, “nutritional assessment (*C*_34_)” and “activities of daily living (*C*_31_)” might influence frailty, and frailty is highly associated with AKI for elderly patients. Therefore, through proper nutrition assessment, a doctor should ascertain the biological status of the elderly patients and mitigate their associated geriatric risk factors that might cause AKI symptoms.

## Discussion

To our knowledge, this study is the first attempt that leverages meta-analysis and an MCDM technique to form the AKI risk assessment framework for the elderly, considering the COVID-19 pandemic. The meta-analysis highlighted the critical role of frailty that might lead to AKI for elderly patients. The present study deepens the understanding of the relationship among the AKI risk factors by using the DEMATEL method to collect clinical knowledge from a group of experienced nephrologists.

### Clinical Practice

This study indicated that the critical risk factors for elderly AKI with frail patients are comorbidity, malignancy, diabetes, creatinine, estimated glomerular filtration rate, and nutritional assessment. This means that these risk factors require special attention compared to the other ones. Based on the findings, for elderly inpatients, doctors should pay special attention to their comorbidities and nutritional assessment, especially for patients with malignant tumors and diabetes. During treatment, doctors should always heed the values of creatinine and glomerular filtration rate for elderly patients. And frailty levels of elderly patients should be monitored or managed by dealing with nutritional treatment and improving their daily living activities. Therefore, doctors can establish a preliminary observation index and then design a series of preventive guidelines to reduce AKI risk for the elderly. The reason for this is that AKI inpatients are high-risk, as a delayed diagnosis in these patients may lead to irreversible kidney damage, for which we have only a few treatment modalities.

In this study, we considered laboratory values as a crucial aspect. However, we could not clarify whether the laboratory examinations were conducted at admission, during the hospital stay, or at discharge. A previous study indicated the disparity of diagnoses that may occur between admission and discharge in hospitals (42). These discrepancies may lead to unexpected clinical examinations, inappropriate treatments, or delayed care to patients. There should be a warning mechanism to identify the high AKI risk elderly patients at each stage; the proposed DEMATEL framework might be a plausible tool to meet this end.

### Comparative Analysis With COVID-19 Risk Factor

**Figure 3:**
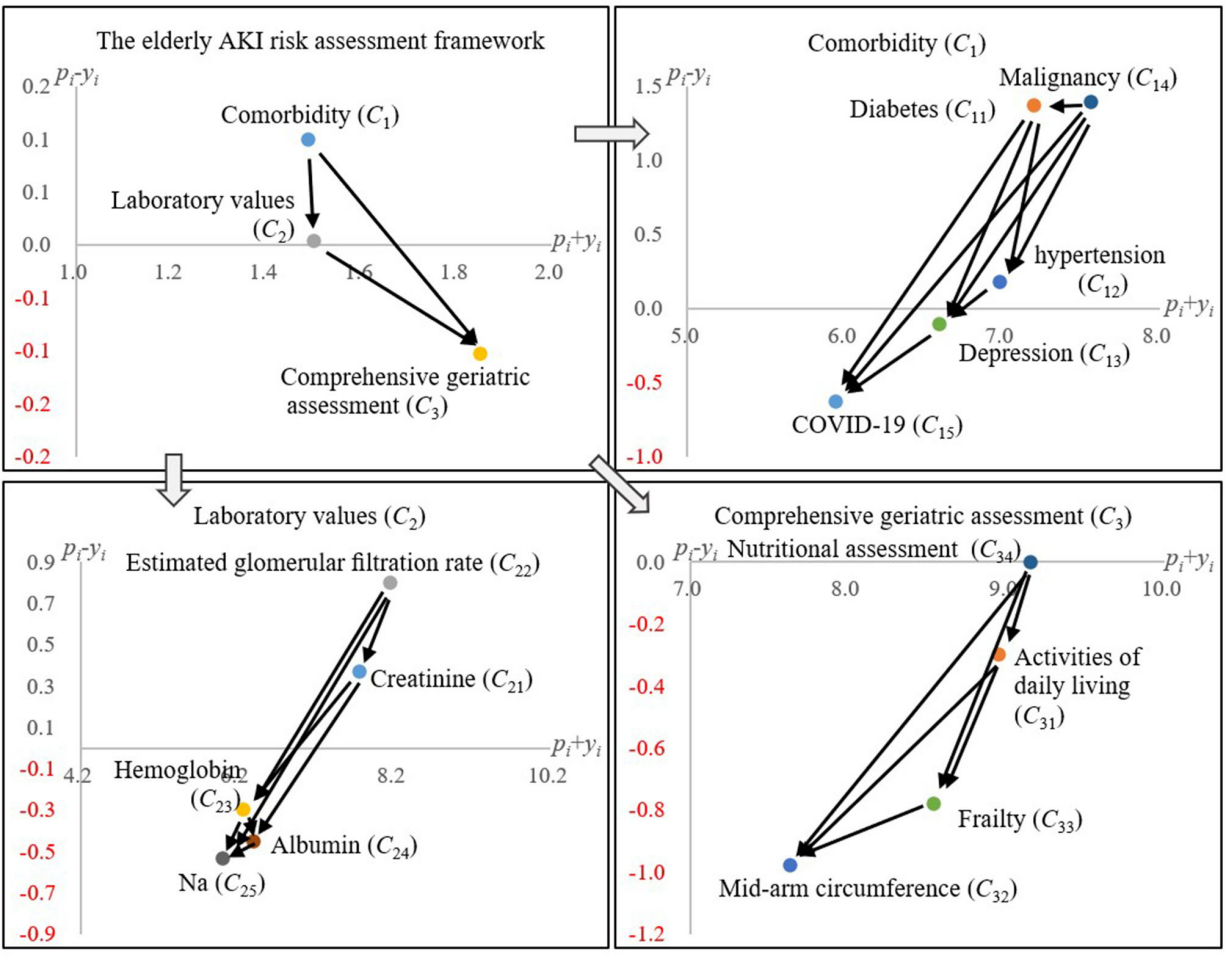
The influential network-relation diagram with COVID-19 risk factor.

For the risk assessment of elderly patients with COVID-19, we also collected the clinical experience of eight nephrologists, and their statistical confidence level is 97.638% (the gap error is 2.361%). The influence relationship among risk factors is shown in Figure 3. Comparing the results of Figures 2, 3, the structure of the influence relationship of all risk factors has not changed; the only difference is that in the comorbidity aspect (*C*_1_), COVID-19 is affected by all other comorbidity factors [i.e., Malignancy (*C*_14_), Diabetes (*C*_11_), Hypertension (*C*_12_), and Depression (*C*_13_)]. In other words, elderly AKI patients generally have some common comorbidities. Later, if they are also confirmed with COVID-19, these comorbidities will affect their treatment process and doctors' treatment methods. Some studies have also pointed out that the elderly and people with chronic diseases will also increase the risk of COVID-19 and death (43, 44), e.g., malignancy, diabetes, and hypertension (45–47).

### Methodological Considerations

From the methodological perspective, this study has several limitations. First, only a few studies discussed the relationship between AKI and the vulnerability of the elderly through quantitative methods. Therefore, this study mainly refers to three studies as the evidence base for establishing an AKI risk assessment framework for the elderly. Second, elderly AKI has many risk factors; it is difficult to comprehensively evaluate the interdependence among all risk factors. Therefore, in a limited aging assessment framework, this study visualizes the interdependent structure among the crucial AKI risk factors without considering other risk factors (e.g., heart failure, infection, and polypharmacy). Third, this study focuses on elderly AKI patients and only collects the nephrologists' clinical experience without considering the experience of doctors in other professions. Finally, the small sample sizes in doctors from the same hospital in Taipei might bias the results. In the first phase, we collected 10 questionnaires from the doctors without considering the COVID-19 risk factor, and in the next stage (i.e., including the COVID-19 risk factor) only eight of the 10 doctors provided valid opinions. All the doctors have limited experience on handling COVID-19 elderly patients. Further investigations with larger study sample sizes would make the results more robust.

## Conclusions And Remarks

Previous studies mainly relied on statistics to examine the risk factors associated with AKI for elderly patients. However, most statistical models have to presume an independent relationship among the factors. The influential relationship is relatively under-explored. This study proposes the DEMATEL method to examine how various risk factors affect each other interactively. The description of these effects may help establish a complete decision-making model. Therefore, the AKI diagnosis and treatment process of the elderly considers the causal relationship between attributes, which allows doctors to avoid the decision problem of treating symptoms rather than diseases. Also, elderly patients are more vulnerable to COVID-19. Thus, this study's key strengths are twofold: (1) it explored the influential relationship among the crucial risk factors of AKI for elderly patients from doctors' clinical experience and (2) provided systematic guidance to manage elderly patients' AKI risk with or without COVID-19. Finally, we suggested that more AKI studies should be conducted for elderly patients with or without COVID-19 to provide more comprehensive and accurate results.

## Data Availability Statement

The original contributions presented in the study are included in the article/supplementary material, further inquiries can be directed to the corresponding author/s.

## Ethics Statement

Ethical review and approval was not required for the study on human participants in accordance with the local legislation and institutional requirements. The patients/participants provided their written informed consent to participate in this study.

## Author Contributions

Y-CC and T-HT conducted the study and drafted the manuscript. J-YC participated in the design of the study. K-YS and C-WC conceived of the study and participated in its design and coordination. All authors read and approved the final manuscript.

## Conflict of Interest

The authors declare that the research was conducted in the absence of any commercial or financial relationships that could be construed as a potential conflict of interest.
